# Metastrongyloid Infection with *Aelurostrongylus abstrusus*, *Troglostrongylus brevior*, *Oslerus rostratus* and *Angiostrongylus chabaudi* in Feral Cats from the Canary Islands (Spain)

**DOI:** 10.3390/ani13132168

**Published:** 2023-06-30

**Authors:** Katherine García-Livia, Ricardo Reyes, Virginia Amaro-Ramos, Edgar Baz-González, Natalia Martin-Carrillo, Eligia Rodríguez-Ponce, Pilar Foronda

**Affiliations:** 1 Instituto Universitario de Enfermedades Tropicales y Salud Pública de Canarias (IUETSPC), Universidad de La Laguna (ULL), Avda. Astrofísico F. Sánchez s/n, 38203 San Cristóbal de La Laguna, Spain; kgarcial@ull.edu.es (K.G.-L.); rreyesro@ull.edu.es (R.R.); vamarora@ull.edu.es (V.A.-R.); ebazgonz@ull.edu.es (E.B.-G.); nmartinc@ull.edu.es (N.M.-C.); 2 Department Obstetricia y Ginecología, Pediatría, Medicina Preventiva y Salud Pública, Toxicología, Medicina Legal y Forense y Parasitología, Universidad de La Laguna (ULL), Avda. Astrofísico F. Sánchez s/n, 38203 San Cristóbal de La Laguna, Spain; 3 Programa de Doctorado en Biodiversidad y Conservación, Universidad de La Laguna (ULL), Avda. Astrofísico F. Sánchez s/n, 38203 San Cristóbal de La Laguna, Spain; 4 Department Bioquímica, Microbiología, Biología Celular y Genética, Universidad de La Laguna (ULL), Avda. Astrofísico F. Sánchez s/n, 38203 San Cristóbal de La Laguna, Spain; 5 Programa de Doctorado en Ciencias Médicas y Farmacéuticas, Desarrollo y Calidad de Vida, Universidad de La Laguna (ULL), Avda. Astrofísico F. Sánchez s/n, 38203 San Cristóbal de La Laguna, Spain; 6 Department Patología Animal, Facultad de Veterinaria, Universidad de Las Palmas de Gran Canaria (ULPGC), Trasmontaña, Arucas, s/n, 35413 Las Palmas, Spain; eligia.rodriguezponce@ulpgc.es

**Keywords:** *Angiostrongylus chabaudi*, *Oslerus rostratus*, *Troglostrongylus brevior*, *Aelurostrongylus abstrusus*, metastrongylids, lungworms, feral cats, La Gomera, Canary Islands

## Abstract

**Simple Summary:**

Lung parasitosis in cats can be asymptomatic or produce a variety of symptoms ranging from mild to severe and potentially life-threatening. In Europe, lungworms have been studied mainly in domestic cats and European wildcats. However, studies on cats in Spain are scarce, especially in the Canary Islands, where *Aelurostrongylus abstrusus* has been the only lungworm species documented to date in feral cats. The present study was conducted in order to provide new epidemiological data on lungworm infections in feral cats in the Canary Archipelago. More than half of the cats analyzed in this study presented pulmonary nematodes, identifying a total of four metastrongylid species, namely *A. abstrusus*, *Troglostrongylus brevior*, *Oslerus rostratus* and *Angiostrongylus chabaudi*. Veterinary practitioners should consider these parasites as possible causes of respiratory pathologies in cats in the Canary Islands. In addition, control measures should be carried out to prevent the transmission of these respiratory parasites.

**Abstract:**

Lungworms are a major cause of feline respiratory disease, frequently underdiagnosed due to its presentation of symptoms being similar to that of other feline respiratory pathologies. Epidemiological data about these nematodes are scarce in the Canary Islands (Spain). Given the veterinary importance of these parasites, the aim of the present study was to improve the current epidemiological knowledge of the lungworm species that could be affecting feral cats in this archipelago. A total of 29 feral cats from La Gomera were analyzed. The respiratory tract of each animal was inspected and the nematodes obtained were identified by morphological keys and molecular techniques. Metastrongylids were detected to be widely distributed throughout the island with a prevalence of 55.2% (16/29). The species *Aelurostrongylus abstrusus*, *Troglostrongylus brevior*, *Oslerus rostratus* and *Angiostrongylus chabaudi* were identified. Also, coinfections with *A. chabaudi* and *O. rostratus* were detected in two animals. The present study shows a high diversity of lungworms in feral cats in La Gomera, with the first report of *A. chabaudi* and *T. brevior* for the Canary Archipelago and the first citation of *A. chabaudi* in cats for Spain. The wide distribution and high prevalence found in this study indicate a high risk of exposure to pulmonary infections in cats.

## 1. Introduction

Lungworms are among the most important parasites affecting the respiratory system of cats worldwide [1,2]. In the last decade, these respiratory nematodes have gained the attention of the veterinary community because of the increasing number of infections reported in cats from many European countries, becoming a primary cause of respiratory disease in clinical practice and feline parasitology [2,3,4]. In fact, a study with more than 1900 cats from 12 European countries estimated that lungworms are the second-most frequently diagnosed group of parasites in cats after ascarid worms [3].

The transmission of respiratory nematodes in small felids can occur via the transmammary route [5] or by ingestion of gastropod intermediate hosts and, more frequently, by predation of paratenic hosts such as lizards, frogs, birds and rodents, among others, which are all part of the diet of cats [6]. Consequently, feral and outdoor cats are considered to be at higher risk of infection with these parasites than indoor cats, although indoor cats are also susceptible to infection, especially when the intermediate or paratenic hosts have easy access to the dwelling place [4,7,8,9,10]. Once an animal ingests the infective larvae, they migrate through the body of the animal and reach the respiratory tract, where they evolve into adulthood. Depending on the lungworm species, the adult stage may be located in the trachea, pulmonary arteries, alveoli, alveolar ducts, bronchi or bronchioles [11,12,13,14,15]. Later, adult females release eggs that, after hatching, produce L1 larvae and pass into the upper respiratory tract to be swallowed and excreted into the environment through feces [11,12,13,14,15].

The signs of lungworm infections range from mild to severe, with a variety of clinical signs and symptoms such as persistent cough, wheezing, sneezing, runny nose and labored breathing, among others [16]. All these symptoms are very similar to other feline respiratory pathologies, for example, feline asthma, allergic bronchitis or pneumonia; therefore, feline lungworms can be frequently underdiagnosed [2,16,17,18]. The anatomical location of adult nematodes, together with factors such as the age and immune status of the animal, the parasitic species and the degree of infection, may further exacerbate the severity of clinical symptoms, especially in cases of coinfection with multiple nematode species, thus compromising the life of the animal [1,19,20]. 

The most well-known and widespread lungworm reported in felids is the metastrongyloid *Aelurostrongylus abstrusus*, which is considered the most prevalent species in domestic cats and is endemic to many European countries [3,11,15,21,22]. In addition, other species such as the capillaroid *Capillaria aerophila* (syn. *Eucoleus aerophilus*) and the metastrongyloids *Troglostrongylus* spp., *Oslerus rostratus* and *Angiostrongylus chabaudi*, among others, have also been cited to affect cats [1,2,3,4,13,16,20,23].

Epidemiological surveys of lungworm species affecting cats in Spain recorded a general prevalence ranging from 0.3 to 24.1%, with the highest prevalence values found in stray cats studied by necropsies [10,24,25]. In addition, a recent European study on domestic cats showed that 6.5% of these animals in Spain were infected with lungworms [3]. The species *A. abstrusus*, *T. brevior* and *O. rostratus* are metastrongylid species frequently detected in cats in Spain and have been cited to affect feral, stray and owned cats from mainland Spain and the Balearic Islands [3,24,25,26,27,28].

In the Canary Islands (Spain), an archipelago composed of eight islands and islets located close to the northwest side of Africa, the overall prevalence of lungworms reported in cats in the available studies ranged from 8.9 to 28.3% [29,30,31,32]. In these studies, the species *A. abstrusus* and *O. rostratus* were cited as infecting feral cats from Gran Canaria and domestic cats from Tenerife, respectively [29,30,31,32]. In light of these reports and considering the respiratory pathology that these parasites can produce, the aim of this study was to improve the current epidemiological knowledge on the distribution of feline lungworms in the Canary Islands. For this purpose, the present study analyses the distribution, prevalence and identification of the metastrongylid species affecting feral cats from the Canary Island of La Gomera.

## 2. Materials and Methods

### 2.1. Sample Collection

Between June 2021 and July 2022, a total of 29 feral cats (*Felis catus*) from La Gomera, captured and provided already sacrificed by Gestión y Planeamiento Territorial y Medioambiental (GESPLAN) staff, with the authorization of Gobierno de Canarias (expedient number 2022/25073) and Excmo. Cabildo Insular de La Gomera (expedient numbers 1872/2021 and 1821/2022), were analyzed at the Instituto Universitario de Enfermedades Tropicales y Salud Pública de Canarias (Table S1).

**Figure 1:**
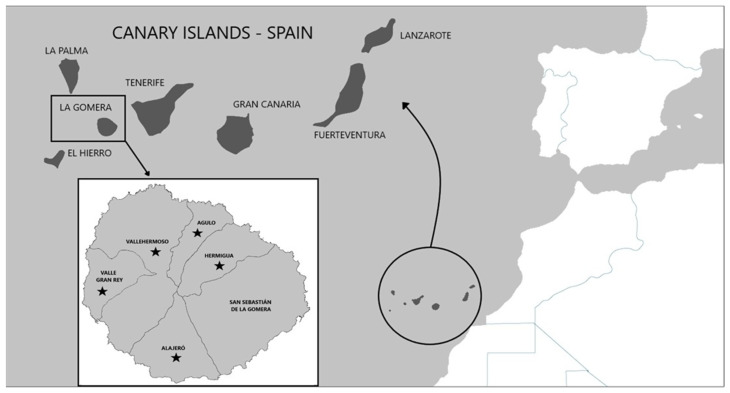
Geographical location of the Canary Islands and the municipalities of the island of La Gomera that were analyzed. Black stars represent the location of the feral cats that were positive for lungworms.

The animals used in this study were obtained in the municipalities of Vallehermoso (*n* = 16), Valle Gran Rey (*n* = 6), Hermigua (*n* = 3), Agulo (*n* = 2), San Sebastián de La Gomera (*n* = 1) and Alajeró (*n* = 1) (Figure 1).

After dissection, the lungs and heart of each animal were examined for adult lungworms using a Leica M80 (Leica Mikrosysteme Vertrieb GmbH, Wetzlar, Germany) stereoscope. The nematodes obtained were washed in sterile phosphate-buffered saline (PBS) and preserved in 70% ethanol. The central part of a random selection of nematodes was reserved for DNA extraction and the anterior and posterior ends were clarified in lactophenol for morphological identification according to the available keys [7,23,33,34,35,36]. Digital images and measurements in µm were taken using an optical Leica DM2500 (Leica Microsystems, Heerbrugg, Switzerland) microscope and Leica LAS AF 4.12 software, respectively. In addition, a portion of positive lung tissue was fixed directly for histological analysis and the rest of the lung tissue remained frozen until molecular analysis.

### 2.2. Histological Analysis

To determine the existence of lesions, lungs positive for metastrongylids were taken randomly and processed for histological analysis following the standard protocols. Briefly, samples were fixed in 10% formalin solution and embedded in Paraplast^®^. Sections of a 5 μm thickness were obtained from each sample with a microtome (Shandon Finesse 325, ThermoFisher Scientific, MA, USA). The sections were stained with hematoxylin-erythrosine and Masson-Trichrome staining for tissue structure evaluation. Sections were analyzed using light microscopy (Leica DM 4000B, Leica, Barcelona, Spain) and images were captured with a camera (Leica DFC 300FX, Leica, Barcelona, Spain).

### 2.3. DNA Isolation

A portion of a lungworm-positive lung and the middle part of randomly selected nematodes were homogenized in 250 µL of a lysis solution containing 30 mM Tris-HCl (pH 8.0), 10 mM EDTA and 0.4% SDS. Then, 3 µL of proteinase K (20 ng/µL) was added to the samples and incubated at 56 °C overnight. After the inactivation of proteinase K, DNA was extracted according to the method described by Lopez et al. [37]. The quantity and quality of the extracted DNA were determined using a DeNovix DS-11+ spectrophotometer (DeNovix Inc., Wilmington, DE, USA). The DNA was stored at −20 °C until further processing.

### 2.4. PCR Amplification

A fragment of the internal transcribed spacer 2 region (ITS-2) was amplified using the primers NC1 (5′-ACGTCTGGTTCAGGGTTGTT-3′) and NC2 (5′-TTAGTTTCTTTTCCTCCGCT-3′) as previously described by Gasser et al. [38]. PCR reactions were performed in a total volume of 25 μL, including 10× buffer Mg^2+^ free (Bioline, London, UK), 2.5 μL of each dNTP (10 mM), 1 μL of each primer (12.5 ng/mL), 0.125 μL of Biotaq polymerase (5 U/mL) (Bioline, London), 0.75 mM MgCl_2_, 20–50 ng of genomic DNA and water. The ITS-2 fragment was amplified using the following cycling conditions: 2 min at 94 °C, followed by 35 cycles of denaturation at 94 °C for 1 min, annealing at 58 °C for 1 min and extension at 72 °C for 1 min, with a final extension step at 72 °C for 5 min. All PCR products were resolved on 1.5% agarose gel.

### 2.5. Sequencing and Phylogenetic Analysis

Positive amplicons were sequenced in both directions at Macrogen Inc. (Madrid, Spain). The obtained nucleotide sequences were edited with the MEGA X program [39] and subsequently aligned with other metastrongylid sequences using the Clustal-W program included in MEGA X. Minor corrections to increase the aligned sequence similarity and improve the inferences on any positional homology were then made by hand. A BLAST search was performed to elucidate any homologies or similarities with the sequences previously published in the GenBank database. Molecular identification was achieved using phylogenetic analysis through the neighbor-joining distance method with the p-distance model [40] and the maximum likelihood method with the Kimura 2-parameter model [41], both with at least 1000 bootstrap replications. *Toxocara cati* (Acc. Number: JN391472) was used as the outgroup.

## 3. Results

### 3.1. Morphological Analysis 

**Figure 2:**
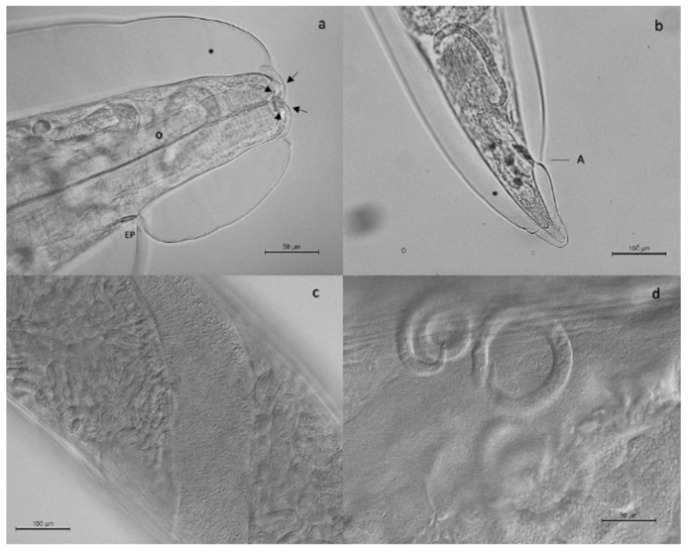
Adult female of *Troglostrongylus brevior*. The cephalic region (**a**) presented an oral opening surrounded by pairs of small papillae (arrows). The excretory pore (EP) was situated in the first third of the esophagus (O). The caudal region (**b**) was short and conical, note the anus (A). In both cephalic and caudal regions, the cuticle was inflated and folded (*). The gravid uterus (**c**,**d**) contained larvated eggs and developed larvae.

**Figure 3:**
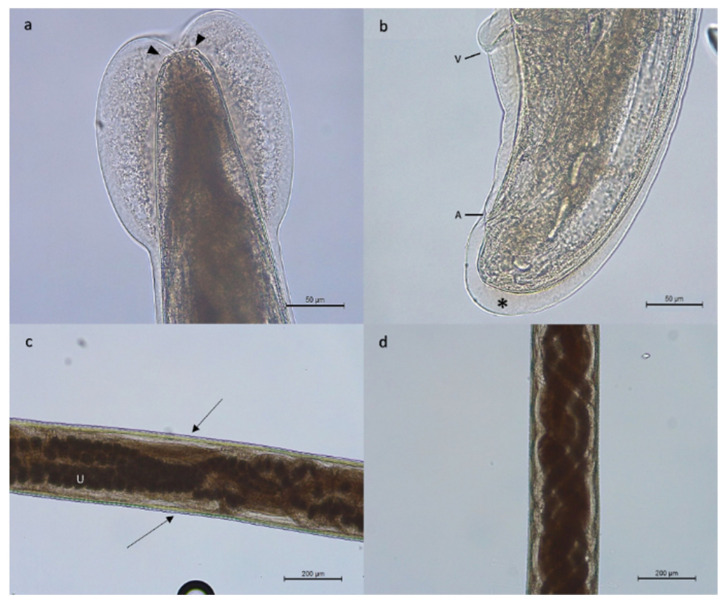
Adult female of *Angiostrongylus chabaudi*. Cephalic region (**a**) showing the cephalic vesicle, with a circular oral aperture surrounded by labial papillae (arrowheads). Caudal region (**b**) ventrally curved, showing the anal opening (A), the vulvar opening (V) and an inflated cuticle (*). Note the vulva is anterior to the anus. Middle of the body (**c**,**d**) showed transversal striation of the cuticle (arrows) and the intestine intertwined with the genital tube. Note the uterus (U) filled with eggs.

**Figure 4:**
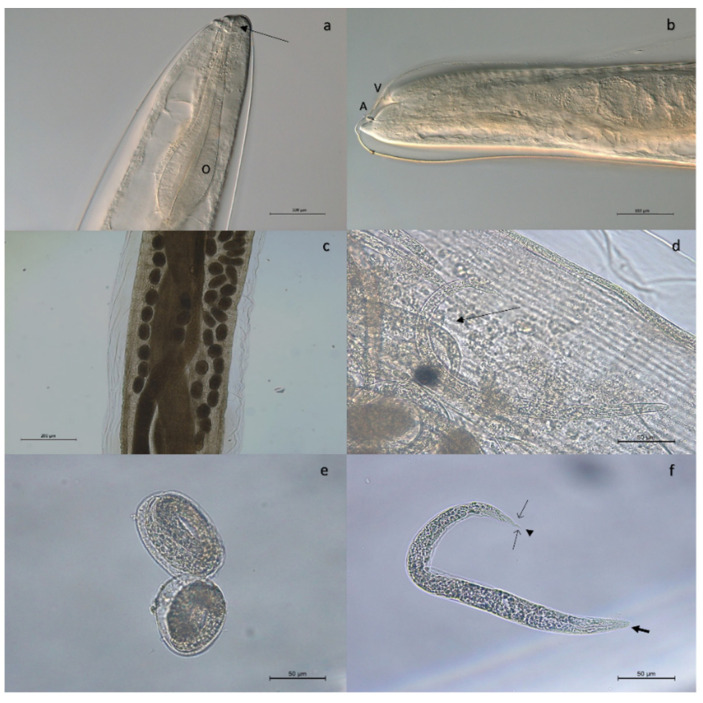
Adult female of *Oslerus rostratus*. The cephalic region (**a**) showed a retracted oral opening (arrow) and a club-shaped esophagus (O). Caudal region (**b**) surrounded by three protuberances, one of them longer than the other two giving the appearance of a tail. Note the vulva (V) located near the anus (A). The intestine (**c**) was dark brown and tubular, running through the body coiled around the uteri full of eggs (**e**) and developed larvae (**d**,**f**). The first-stage larvae (**f**) presented a rounded head with a central oral opening surrounded by a cuticular ring (bold arrow). The tail showed a deeper notch on the ventral side and a shallower one on the dorsal side (light arrows) with a minute cuticular spine at the proximal edge of the dorsal notch (arrowhead).

Adult stages of pulmonary nematodes were detected in the lungs of 16 out of 29 feral cats analyzed (55.2%) from 5 out of 6 municipalities of La Gomera, concretely, Vallehermoso (43.7%; 7/16), Valle Gran Rey (50%; 3/6), Hermigua (66.6%; 2/3), Agulo (100%; 2/2) and Alajeró (100%; 1/1) (Figure 1). The mean intensity was 9.0 worms per animal, with an intensity range of 1 to 69. Gross tissue examination and morphological analysis allowed us to identify adult females of three metastrongylid species: *T. brevior* (Figure 2), *A. chabaudi* (Figure 3) and *O. rostratus* (Figure 4), while no males were detected. Additionally, it was not possible to morphologically identify two nematodes due to the damage to their ends suffered during isolation.

Adult females of *T. brevior* were 6–13 mm in length and 330–430 µm in width. Females of *A. chabaudi* were 21–22 mm in length and 260–270 µm in width. In the case of *O. rostratus*, worms were long and slender with a smooth integument. It was not possible to measure the total length of the females obtained, as they suffered damage during extraction due to being embedded in the peribronchial tissue surrounded by a fibrous tissue capsule.

### 3.2. Histological Evaluation

**Figure 5:**
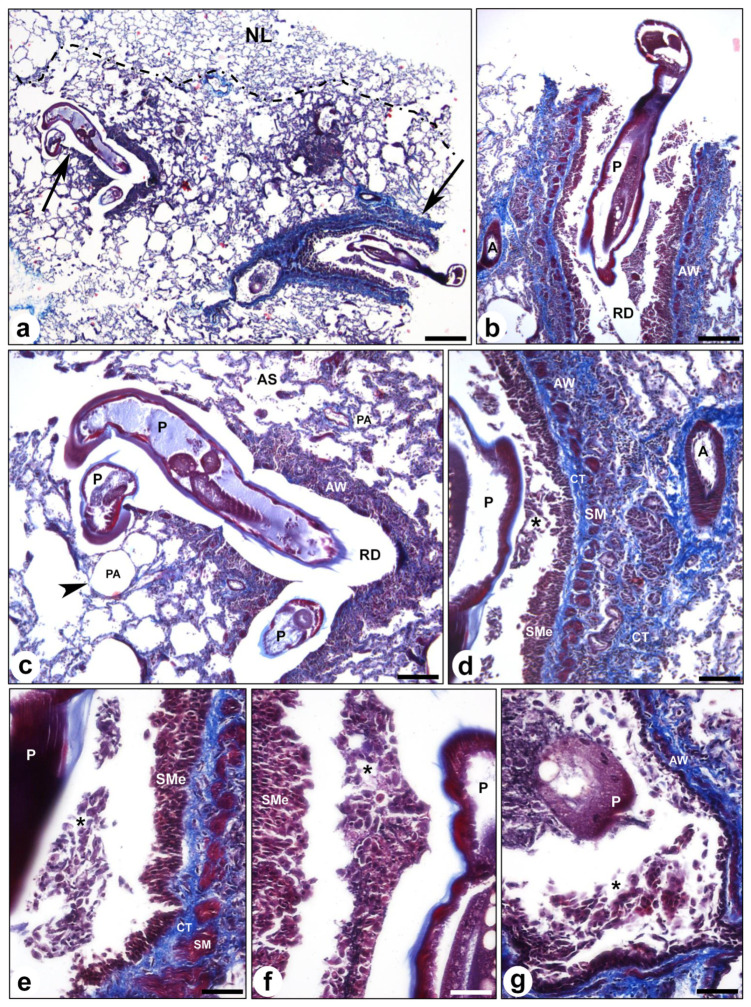
Histological evaluation. Representative images showing pulmonary lesions caused by *Oslerus rostratus* infection. (**a**) Panoramic image showing an area of the lung with preserved tissue architecture (above the dashed line) and an area affected by the infestation in which the presence of two granulomatous lesions (arrows) containing the parasite inside can be observed. (**b**,**c**) Magnification images of the lesions at different levels of the respiratory tract with alveolar dilatation (arrowhead). (**d**–**g**) Magnification images showing hypertrophy of the airway wall in one of the lesions with the presence of intramural inflammatory infiltrate (*) as well as in the lumen of the duct surrounding the parasite. Note the extensive wall fibrosis (CT) with smooth muscle hyperplasia (SM) as well as the presence of squamous metaplasia (SM) of the respiratory epithelium. A: artery, AS: alveolar sac; AW: airway wall; CT: connective tissue; NL: normal lung; P: parasite; PA: pulmonary alveoli; RD: respiratory duct; SM: smooth muscle; SMe: squamous metaplasia; scale bars: (**a**) 1 mm, (**b**,**c**) 500 μm, (**d**) 250 μm, (**e**–**g**) 100 μm. Masson-Trichrome staining.

The analyzed samples showed loss of normal lung tissue architecture with distortion of the alveolar structure and fibrosis in those areas where the parasite, *O. rostratus*, was located (Figure 5). Two granulomatous lesions were observed in the lung parenchyma containing the parasite, inside the respiratory ducts (Figure 5a–c). The lesions were characterized by airway wall hypertrophy with epithelial cell proliferation and squamous metaplasia instead of normal ciliated pseudostratified epithelium, an increase in connective tissue and smooth muscle hyperplasia (Figure 5d–g). Inflammatory infiltrate was also observed in the airway wall and around the parasite, with numerous macrophages, lymphocytes and eosinophils (Figure 5e–g).

### 3.3. Molecular Examination

The results obtained in the molecular analysis confirmed the morphological results obtained, also identifying the species *T. brevior*, *A. chabaudi* and *O. rostratus*. Additionally, two nematodes that were not possible to identify morphologically due to damage to their ends during isolation were identified as *A. abstrusus*. The molecular analysis of the lung samples also confirmed the identity of previously obtained species and also detected coinfections with *A. chabaudi* and *O. rostratus* in two animals.

A total of seventeen nucleotide sequences were obtained. *Troglostrongylus brevior* was confirmed in three cats (isolates 101P, 103P and 1603N), displaying 99–100% homology with others *T. brevior* sequences (GenBank: MK675815, MH537789). *Oslerus rostratus* was identified in seven cats (isolates 202NP, 1607N, 603NPN2, 603NPN4, 703N, 905N and 903NPP), displaying 99% homology with an *O. rostratus* sequence (GenBank: KP987220). *Angiostrongylus chabaudi* was identified in five cats (isolates 204N, 702N, 903NPN, 601P and 603NPP), displaying 99–100% homology with other *A. chabaudi* sequences (GenBank: KU521522, MH899656). *Aelurostrongylus abstrusus* was identified in two cats (isolates 104N and 906), displaying 99–100% homology using BLAST with *A. abstrusus* sequences (GenBank: MK675816, MH779463). The obtained nucleotide sequences generated in this study are available in the GenBank database under the accession numbers OP858709-OP858725.

**Figure 6:**
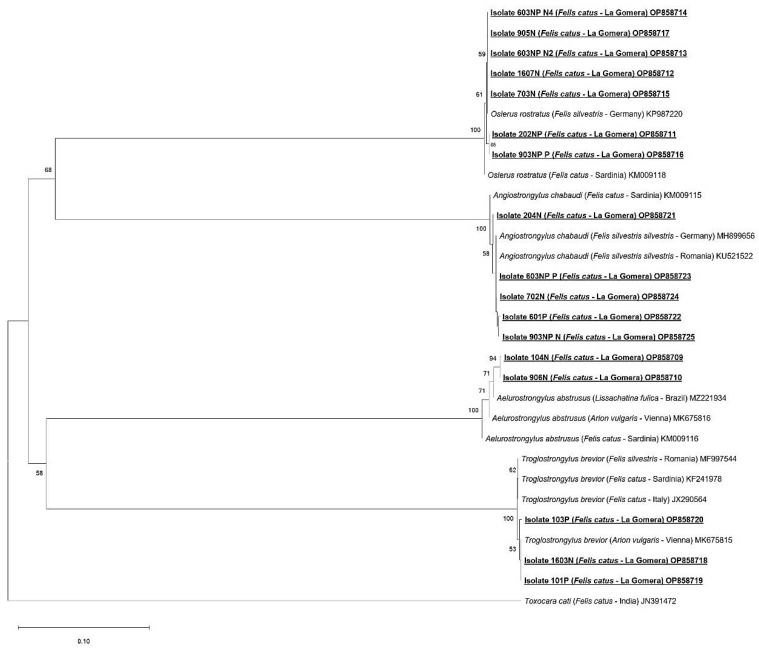
Phylogenetic analysis, based on 358 bp fragment of the internal transcribed spacer 2 (ITS-2), using the neighbor-joining distance method and p-distance model with 1000 bootstrap replications. New sequences obtained in this study are typed in bold, underlined text. *Toxocara cati* was used as the outgroup.

**Figure 7:**
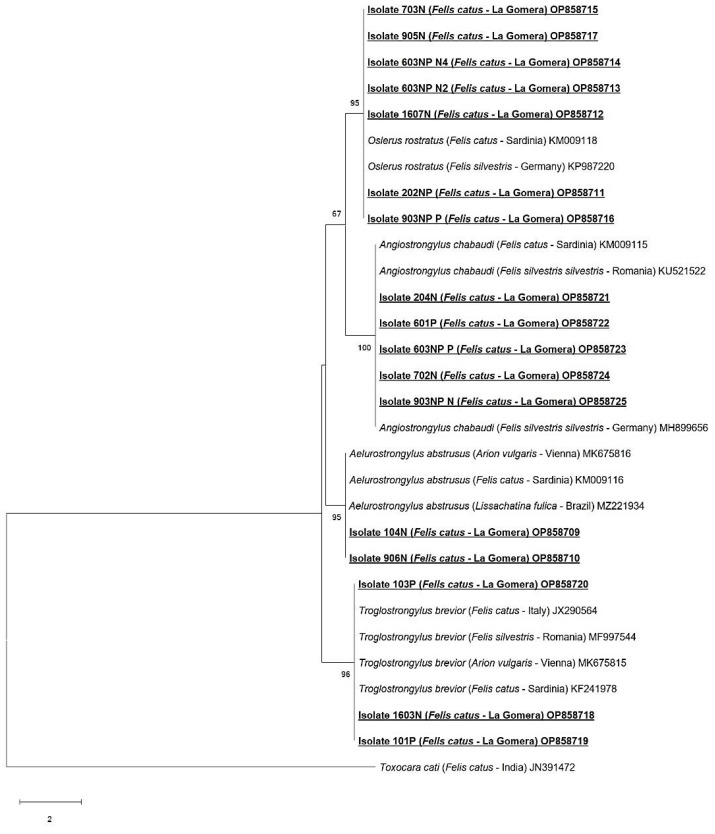
Phylogenetic analysis, based on 358 bp fragment of the internal transcribed spacer 2 (ITS-2), using the maximum likelihood method and Kimura 2-parameter model with 1000 bootstrap replications. New sequences obtained in this study are typed in bold, underlined text. *Toxocara cati* was used as the outgroup.

The results of the neighbor-joining (Figure 6) and maximum likelihood (Figure 7) analyses based on 358 bp of the alignment showed similar results, confirming the identity of the four metastrongylid species detected by BLAST. In both phylogenetic analyses, the sequences from feral cats obtained in this study were grouped into the *A. abstrusus*, *T. brevior*, *O. rostratus* and *A. chabaudi* clades with high bootstrap values (95–100%).

In this study, the cardio-pulmonary species *O. rostratus* and *A. chabaudi* were the most frequently detected species, with an occurrence of 27.6% (8/29) and 17.2% (5/29), respectively, followed by *T. brevior* (10.3%; 3/29) and *A. abstrusus* (6.9%; 2/29) (Table 1). Of the 16 feral cats positive for cardio-pulmonary parasites, 14 animals presented single infections with *O. rostratus* (*n*= 6), *A. chabaudi* (*n* = 3), *T. brevior* (*n* = 3) and *A. abstrusus* (*n* = 2). Coinfections with *A. chabaudi* and *O. rostratus* were detected in two animals. 

## 4. Discussion

In recent years, the parasitosis of the respiratory system of felids has attracted the attention of the veterinary community due to their great geographical expansion and the epizootiological and biological characteristics that have been discovered in these nematodes [2,3,4].

In Europe, these pulmonary nematodes have been studied mainly in wildcats and domestic cats [15]. In the case of Spain, most available epidemiological investigations are based on wild animals such as wolves and foxes. However, studies related to cats from this region are scarce [42,43]. Therefore, the present study contributes to the knowledge of the epidemiology of the feline cardio-pulmonary nematodes affecting feral cats in Spain, providing new data on the prevalence and distribution of these parasites in the Canary Archipelago.

These nematodes have been previously cited worldwide, mostly the species *A. abstrusus*, which is frequently reported in cases of infections in domestic cats [1]. Other species, such as the metastrongyloids *T. brevior*, *O. rostratus* and *A. chabaudi*, have gained more veterinary relevance given the increase in the number of reports documented in wildcats and domestic cats, most of them geographically limited to Europe [2,4]. 

In this study, metastrongylid infections were detected in 16 feral cats from the island of La Gomera. A total of four species were successfully identified, concretely, *A. abstrusus*, *T. brevior*, *O. rostratus* and *A. chabaudi*. Up to now, only the species *A. abstrusus* and *O. rostratus* had been reported in feral and domestic cats of other Canary Islands, namely Gran Canaria and Tenerife, respectively [29,30,32].

The general prevalence obtained in our study was 55.2%, higher than the percentage reported in two other studies carried out in the Canary Islands, where 10.4% (5/48) and 28.3% (82/290) were reported in feral cats from Gran Canaria [30,31]. However, the prevalence of metastrongylid infections obtained in our study is similar to previous studies carried out in other insular areas, such as Sri Lanka, where 60% was reported [44]. 

There have been several studies carried out in island regions that have documented the presence of metastrongylid species in wildcats, stray, feral and domestic cats, such as Sri Lanka [44], Hawaii [45], Majorca [25], Ibiza [26], Sicily [23,35], Crete, Mykonos, Skopelos [46,47,48,49], Cyprus [50], Sardinia [51,52,53], Gran Canaria and Tenerife [29,30,31,32], among others. The most common lungworm species reported in these islands are *A. abstrusus*, *T. brevior* and *O. rostratus*. However, the cardio-pulmonary nematode *A. chabaudi* has only been previously reported in an adult cat on the island of Sardinia [51], with our study being the second report of *A. chabaudi* on islands.

Of all metastrongylid species detected in this study, *O. rostratus* was the most frequently detected species, with a 27.6% prevalence, followed by *A. chabaudi* (17.2%), *T. brevior* (10.3%) and *A. abstrusus* (6.9%). These results are in contrast to the previous data for Spain, where *A. abstrusus* was reported as the most prevalent metastrongylid species, with prevalence ranging between 1 and 10.4%, with the highest percentage being obtained for Gran Canaria [24,25,30].

*Aelurostrongylus abstrusus* is a parasite that affects feline lung tissues causing aelurostrongyliasis, a disease of the lower respiratory tract, whose serious infections can lead to verminous pneumonia which can be fatal [54,55,56,57]. This cosmopolitan nematode is the lungworm species most frequently diagnosed in felids, being reported in Europe, South America, Australia, the Middle East, Russia, the Far East, the USA, China and Africa [3,9,22,54,58,59,60,61,62,63,64,65,66,67]. The prevalence of *A. abstrusus* can vary significantly across Europe. It has been reported ranging from 0.4% in Croatia [68] to 43.1% in Albania [69], with Italy being the European country with the second-highest prevelance (26.5%) [70]. In addition, the prevalence rate of this lungworm species may vary within the same country (see [22]), for example, a 1% prevalence of *A. abstrusus* has been reported in feral cats from mainland Spain [24], while a 10.4% prevalence in feral cats from the island of Gran Canaria [30], with this percentage being similar to the prevalence obtained for *A. abstrusus* in our study.

*Troglostrongylus brevior*, after its first description in wild felids from Palestine [7], was reported mostly in wildcats from central Italy [4,13,71,72] until 2010, when it was first detected in domestic cats from Ibiza (Spain) [26]. In the last decade, *T. brevior* has been reported to infect domestic cats from the islands of Sicily and Sardinia in Italy, the islands of Crete, Mykonos and Skopelos in Greece, in the island state of Cyprus [35,46,47,50,73,74] and in continental areas such as Italy [74,75,76,77], Greece [47], Bulgaria [3], Spain [3], Romania [78,79] and Turkey [80]. Therefore, our study constitutes the first report of *T. brevior* in feral cats in the Canary Islands and the northwestern area close to the African continent.

The clinical manifestations of *T. brevior* are similar to those produced by *A. abstrusus*, being potentially fatal in kittens and cats less than one year old [1,35,48,79,81,82]. However, the lesions caused by both species are different: while *T. brevior* is longer in size and inhabits the bronchi and bronchioles of the definitive host, *A. abstrusus* is smaller and is localized in the alveolar ducts and the lung parenchyma of hosts [1,2,35,81]. *Troglostrongylus brevior* is usually reported with a lower prevalence in comparison to *A. abstrusus*. However, in certain regions, its prevalence is higher, as has been shown in ther region in our study and in other regions such as Greece [47], Cyprus [50], Italy [83] and Israel [84].

*Oslerus rostratus*, which can be found in the peri-bronchial tissues and in the bronchial walls, was first described as *Anafilaroides rostratus* in cats from Jerusalem [7]. Later, it was reported in cats from Sri Lanka [85], Hawaii [45], mainland Spain [86], Majorca and Ibiza [25,26], Sicily and Sardinia, [23,52] and Hungary [87]. Recently, *O. rostratus* has been cited in domestic cats from the Canary Island of Tenerife [32], with the present study being the first citation in feral cats. In mainland Spain, *O. rostratus* has been one of the least prevalent metastrongylid species detected in domestic cats, with a 1% prevalence reported [3]. However, in feral cats from the Spanish island of Majorca, *O. rostratus* has been reported with a 24.1% prevalence [25], being similar to that obtained in our study (27.6%).

*Angiostrongylus chabaudi* inhabits the small pulmonary arteries of wildcats and produces severe damage to the vascular system [11]. It was first described in wildcats from central Italy [88]. Later, it was reported in Sardinia [51] and in coinfection with *A. abstrusus* and *T. brevior* in a domestic cat in central Italy [76]. Subsequently, several studies have reported *A. chabaudi* in the European wildcat in Italy [89], Germany [90], Greece [36,91], Romania [92,93], Bulgaria [11] and, more recently, in Bosnia and Herzegovina [94]. Our study constitutes the first report on *A. chabaudi* in cats from Spain.

Although previous cases of coinfection with cardio-pulmonary nematodes in cats have been cited, such as *A. abstrusus*, *T. brevior* and *A. chabaudi* in a domestic cat in Italy [76], *A. abstrusus* and *A. chabaudi* in a wildcat in Bosnia and Herzegovina [94] and *A. abstrusus*, *T. brevior*, *E. aerophilus* and *A. chabaudi* in a wildcat in Greece [95], among others, our study constitutes the first report in cats of a mixed infection caused by *A. chabaudi* and *O. rostratus*. 

Insular regions are particularly vulnerable to the effects of introduced species such as cats. These animals can interfere with the natural dynamics of populations and/or ecosystems and may originate or favor processes such as the introduction of parasites [96]. In the Canary Islands, cats have been introduced for more than 2300 years [97,98,99]. Considering the high diversity of metastrongylid species identified together with the high prevalence obtained in this study, lungworms could have been introduced to La Gomera with the cat at some point in the past.

On the other hand, the possibility should not be discarded that cats living in endemic areas could have traveled with their owners to the Canary Islands and lungworms have found a way to establish themselves in the archipelago, as was proposed in the finding of *Gurltia paralysans*, a parasite endemic to South America, that has been recently reported infecting a domestic cat in Tenerife [100]. In this sense, it is important to note that La Gomera has a high tourist influx of European travelers.

The results obtained in this study show that feral cats from La Gomera may play a role in the dissemination of lungworm species, increasing the risk of infection to both wild and domestic cats with outdoor access. Infection may occur with one or more species in coinfection, which would further aggravate the symptoms and worsen the health status of these animals. More studies are needed to better understand the distribution of these metastrongylid species in the Canary Archipelago. Furthermore, it is necessary to warn the veterinary community of the Canary Archipelago so that they can make a correct diagnosis in clinical cases with respiratory symptoms.

## 5. Conclusions

This study contributes to the knowledge of the epidemiology of lungworm parasites in cats, being the first report on the high diversity of metastrongylid species in feral cats in the Canaries, reporting *A. chabaudi* and *T. brevior* for the first time in this region and *A. chabaudi* in cats in Spain. The diversity of species and the high prevalence obtained indicate that wild and domestic feline populations in this region are exposed to lungworm infections with *A. abstrusus*, *T. brevior*, *A. chabaudi* and *O. rostratus*, which have been detected to be widely distributed on the island. More studies are needed to better understand the epidemiology of these parasites in the Canary Archipelago. Veterinary practitioners should consider these parasites as possible causes of respiratory pathologies in cats in the Canary Islands. Moreover, control measures should be carried out in order to prevent the transmission of these respiratory parasites.

## Figures and Tables

**Table 1:** Metastrongylid species detected in feral cats and location in La Gomera, Canary Islands (Spain).

Location	Feral Cats +/n (P%)	*Aelurostrongylus abstrusus* +/n (P%)	*Troglostrongylus brevior* +/n (P%)	*Angiostrongylus chabaudi* +/n (P%)	*Oslerus rostratus* +/n (P%)
Vallehermoso	7/16 (43.7%)	-	1/16 (6.2%)	2/16 (12.5%)	4/16 (25%)
Valle Gran Rey	4/6 (66.6%)	2/6 (33.3%)	1/6 (16.6%)	1/6 (16.6%)	1/6 (16.6%)
Hermigua	2/3 (66.6%)	-	1/3 (33.3%)	-	1/3 (33.3%)
Agulo	2/2 (100%)			1/2 (50%)	2/2 (100%)
Alajeró	1/1 (100%)	-	-	1/1 (100%)	-
San Sebastián de La Gomera	0/1 (0%)	-	-	-	-
TOTAL	16/29 (55.2%)	2/29 (6.9%)	3/29 (10.3%)	5/29 (17.2%)	8/29 (27.6%)

+/n: positive samples/sample size. (P%): prevalence.

## Data Availability

Not applicable.

## Supplementary Materials

The following supporting information can be downloaded at: https://www.mdpi.com/article/10.3390/ani13132168/s1, Table S1: Detailed information related to the specimens of *Felis catus* captured in La Gomera (Canary Islands, Spain) analysed in this study and the results obtained.
