# Supplementary material for: Metastrongyloid Infection with Aelurostrongylus abstrusus, Troglostrongylus brevior, Oslerus rostratus and Angiostrongylus chabaudi in Feral Cats from the Canary Islands (Spain)
Source: Animals (Basel). 2023 Jun 30;13(13):2168. doi: 10.3390/ani13132168 (PMC10339911; doi:10.3390/ani13132168)
Supplement: Supplementary file 1 [file animals-13-02168-s001.zip › animals-2396407-supplementary.pdf]

Table S1. Detailed information related to the specimens of *Felis catus* captured in La Gomera (Canary Islands, Spain) analysed in this study and the results obtained.

ANIMAL CODE	SEX	MUNICIPALITY	DATE OF CAPTURE	LUNGWORM DETECTION
22021603	Male	Vallehermoso	June 2021	**Positive**
22021604	Female	Vallehermoso	Unknown	Negative
22021605	Male	Vallehermoso	June 2021	Negative
22021606	Male	Vallehermoso	June 2021	Negative
22021607	Hembra	Vallehermoso	May 2021	**Positive**
22021608	Male	Vallehermoso	May 2021	Negative
22022201	female	Vallehermoso	May /2021	Negative
22022202	Male	Vallehermoso	May 2021	**Positive**
22022203	Female	Vallehermoso	August 2021	Negative
22022204	Male	Vallehermoso	May 2021	**Positive**
22033101	Male	Hermigua	October 2021	**Positive**
22033102	Female	Vallehermoso	November 2021	**Positive**
22033103	Male	Valle Gran Rey	November 2021	**Positive**
22033104	Male	Valle Gran Rey	November 2021	**Positive**
22033105	Male	Valle Gran Rey	December 2021	Negative
22090601	Male	Vallehermoso	July 2022	**Positive**
22090602	Male	Vallehermoso	July 2022	**Positive**
22090603	female	Valle Gran Rey	July 2022	**Positive**
22090701	Male	San Sebastián de La Gomera	July 2022	Negative
22090702	Male	Alajeró	July 2022	**Positive**
22090703	Female	Hermigua	July 2022	**Positive**
22090801	Male	Hermigua	July 2022	Negative
22090901	Female	Vallehermoso	July 2022	Negative
22090902	Male	Vallehermoso	July 2022	Negative
22090903	Male	Agulo	July 2022	**Positive**
22090904	Female	Vallehermoso	July 2022	Negative
22090905	Male	Agulo	July 2022	**Positive**
22090906	Female	Valle Gran Rey	June 2022	**Positive**
22090907	Male	ValleGran Rey	June 2022	Negative
